# The preclinical amyloid sensitive composite to determine subtle cognitive differences in preclinical Alzheimer’s disease

**DOI:** 10.1038/s41598-020-70386-3

**Published:** 2020-08-12

**Authors:** Alice Hahn, Young Ju Kim, Hee Jin Kim, Hyemin Jang, Hanna Cho, Seong Hye Choi, Byeong C. Kim, Kyung Won Park, Duk L. Na, Juhee Chin, Sang Won Seo

**Affiliations:** 1 Department of Neurology, Samsung Medical Center, Sungkyunkwan University School of Medicine, 81 Irwon-ro, Gangnam-gu, Seoul, 06351 Republic of Korea; 2 Neuroscience Center, Samsung Medical Center, 81 Irwon-ro, Gangnam-gu, Seoul, 06351 Republic of Korea; 3 Department of Neurology, Gangnam Severance Hospital, Yonsei University College of Medicine, Seoul, 06273 Republic of Korea; 4 Department of Neurology, Inha University School of Medicine, Incheon, 22332 Republic of Korea; 5 Department of Neurology, Chonnam National University Medical School, Gwangju, 61469 Republic of Korea; 6 Department of Neurology, Dong-A Medical Center, Dong-A University College of Medicine, Busan, 49201 Republic of Korea; 7 Stem Cell Regenerative Medicine Institute, Samsung Medical Center, 81 Irwon-ro, Gangnam-gu, Seoul, 06351 Republic of Korea; 8 Samsung Alzheimer Research Center, Samsung Medical Center, 81 Irwon-ro, Gangnam-gu, Seoul, 06351 Republic of Korea; 9 Center for Clinical Epidemiology, Samsung Medical Center, 81 Irwon-ro, Gangnam-gu, Seoul, 06351 Republic of Korea; 10 Department of Health Sciences and Technology, SAIHST, Sungkyunkwan University, 81 Irwon-ro, Gangnam-gu, Seoul, 06351 Republic of Korea; 11 Clinical Research Design and Evaluation, SAIHST, Sungkyunkwan University, 81 Irwon-ro, Gangnam-gu, Seoul, 06351 Republic of Korea

**Keywords:** Alzheimer's disease, Dementia, Neurodegeneration, Neurodegenerative diseases, Alzheimer's disease

## Abstract

Recently, the focus of Alzheimer’s disease (AD) research has shifted from the clinical stage to the preclinical stage. We, therefore, aimed to develop a cognitive composite score that can detect the subtle cognitive differences between the amyloid positive (Aβ+) and negative (Aβ−) status in cognitively normal (CN) participants. A total of 423 CN participants with Aβ positron emission tomography images were recruited. The multiple-indicators multiple-causes model found the latent mean difference between the Aβ+ and Aβ− groups in the domains of verbal memory, visual memory, and executive functions. The multivariate analysis of covariance (MANCOVA) showed that the Aβ+ group performed worse in tests related to the verbal and visual delayed recall, semantic verbal fluency, and inhibition of cognitive inference within the three cognitive domains. The Preclinical Amyloid Sensitive Composite (PASC) model we developed using the result of MANCOVA and the MMSE presented a good fit with the data. The accuracy of the PASC score when applied with age, sex, education, and *APOE ε*4 for distinguishing between Aβ+ and Aβ− was adequate (AUC = 0.764; 95% CI = 0.667–0.860) in the external validation set (N = 179). We conclude that the PASC can eventually contribute to facilitating more prevention trials in preclinical AD.

## Introduction

With the advancement of amyloid-β (Aβ) positron emission tomography (PET), the focus of research on Alzheimer's disease (AD) has shifted from the clinical and symptomatic stages to the preclinical and asymptomatic stages of AD^1^. Consequently, approximately 20–30% of cognitively normal (CN) elderly population appear to be CN individuals with Aβ positivity^2–4^. These CN individuals with elevated amyloidosis are considered to be more vulnerable to AD progression. This can be demonstrated by the subtle cognitive difference between CN individuals with amyloid positivity and those without Aβ biomarkers in the late preclinical stage^5^. Furthermore, several reports have claimed that approximately 25% of Aβ+ CN individuals converts to mild cognitive impairments (MCI) or dementia in approximately 3 years^6,7^.

Although Aβ PET has such advantage of early detection of Aβ+ biomarker, it is usually challenging to obtain a large number of participants with PET data due to the high cost and safety concerns. However, the importance of Aβ biomarker has kept rising as several prevention trials are currently being conducted in preclinical AD with the expectation to target Aβ. Thus, it will be pragmatic and essential for clinicians to predict who might be at high risk of having an Aβ+ biomarker without the help of neuroimaging techniques. As a result, we need to, instead, investigate the distinct neuropsychological features of CN who have elevated Aβ, which may help clinicians predict preclinical AD by reducing screen failures and monitoring the therapeutic efficacy of prevention.

Previously, there were several attempts to investigate the distinct neuropsychological features of Aβ+ CN individuals. However, the results were inconsistent among studies to date. Multiple studies have consistently reported that high amyloid burden in CN adults is associated with poorer performance in episodic memory^8,9^. On the other hand, a large lifespan study of CN adults found association of amyloid deposition with executive functions, but not with memory burden^10^. This discrepancy may be because not many previous studies considered the effects of measurement errors, although there is always a possibility for the presence of measurement errors in psychometrics^11^. In fact, there was a study considered measurement errors using structural equation modeling (SEM) to examine the associations between amyloid burden or white matter hyperintensity and cognition^9^. However, there are still few studies considering the effects of measurement errors in studying biomarkers and cognition together, and there is a need to build more evidence for this methodology in this regard. Accordingly, we used the multiple-indicators multiple-causes (MIMIC) model in our study to examine Aβ related cognitive functions. By using the principle of factor analysis, the MIMIC model can control for these measurement errors to estimate the latent values. That is, MIMIC may empower a composite model to sensitively detect subtle cognitive differences between Aβ+ and Aβ− in CN elderlies. However, to our knowledge, no Aβ+ CN studies have yet applied this factor structure method to develop a cognitive composite model for identifying preclinical AD in the elderly population.

In the present study, we aimed to determine if there are any distinct cognitive domains and cognitive measures of Aβ+ CN using the MIMIC model, which may yield lower background noise in measurements. We also developed the Preclinical Amyloid Sensitive Composite (PASC) model, a composite model that precisely distinguishes cognitive differences between Aβ+ and Aβ− in CN individuals. Then, we computed the PASC score employing the PASC model we developed. Finally, we validated the PASC score along with age, sex, education, and *APOE ε*4 to distinguish between Aβ+ and Aβ− CN in an external validation set. Considering that subtle deficits in episodic memory and executive functions appear to be critical in preclinical AD due to their strong association with AD progression^12^, we hypothesized that the PASC score consisting of memory and executive functions might differentiate Aβ+ CN from Aβ− CN with adequate accuracy.

## Results

### Demographic characteristics of participants

The demographic and neuropsychological characteristics of the study participants are presented in Table 1. The overall mean age of the participants was 69.9 years. Among the 423 participants, 75 were Aβ+ (17.7%). The frequency of *APOE ε*4 carriers was 24.6%. The Aβ+ group was significantly older than the Aβ− group (71.5 ± 6.8 years vs. 69.5 ± 8.4 years, *p* < 0.05). The Aβ+ group also displayed a higher percentage of *APOE ε*4 carriers compared with the Aβ− group (58.0% vs. 17.5%, *p* < 0.001). However, the two groups did not significantly differ in education level and proportion of female participants.

**Table 1:** Demographic and neuropsychological characteristics of the study participants^a^.

	Development set			Validation set		
	All (N = 423)	Aβ− (N = 348)	Aβ+ (N = 75)	All (N = 179)	Aβ− (N = 150)	Aβ+ (N = 29)
**Demographics**^b^						
Age, years**	69.9 (8.1)	69.5 (8.4)	71.5 (6.8)	69.4 (8.2)	68.6 (8.2)	73.7 (6.8)
Education, years	11.8 (4.8)	11.9 (4.8)	11.3 (4.5)	12.0 (4.4)	12.1 (4.5)	11.9 (4.1)
Female, N (%)	267 (63.1)	219 (62.9)	48 (64)	108 (60.3)	91 (60.7)	17 (58.6)
*APOE* ε4 carrier N (%)^d^**	97 (24.6)	57 (17.5)	40 (58.0)	38 (22.8)	24 (17.1)	14 (51.9)
**Neuropsychological tests**^c^						
Attention						
Digit span forward	6.3 (1.4)	6.3 (1.4)	6.2 (1.3)	6.5 (1.5)	6.5 (1.5)	6.6 (1.4)
Digit span backward	4.1 (1.3)	4.1 (1.4)	4.0 (1.1)	4.4 (1.5)	4.4 (1.5)	4.5 (1.7)
Language						
K-BNT	48.6 (6.7)	48.8 (6.6)	47.7 (7.2)	50.2 (5.3)	50.3 (5.3)	49.2 (5.5)
Visuospatial functions						
RCFT copy	32.7 (3.6)	32.7 (3.7)	32.5 (3.1)	32.9 (2.7)	32.9 (2.6)	32.7 (3.2)
CDT	2.8 (0.5)	2.8 (0.5)	2.8 (0.4)	2.9 (0.4)	2.9 (0.4)	2.9 (0.3)
Memory						
SVLT-E immediate recall	21.4 (4.6)	21.6 (4.6)	20.4 (4.4)	21.7 (4.4)	22.0 (4.4)	19.9 (4.2)
SVLT-E delayed recall	7.0 (2.1)	7.1 (2.1)	6.4 (2.0)	7.1 (2.0)	7.3 (2.0)	6.0 (1.7)
SVLT-E recognition	21.2 (2.0)	21.3 (1.9)	20.8 (2.0)	21.4 (1.6)	21.5 (1.6)	20.8 (1.4)
RCFT immediate recall	14.9 (7.2)	15.3 (7.2)	13.1 (7.4)	16.4 (6.6)	16.7 (6.4)	14.8 (7.1)
RCFT delayed recall	14.8 (6.8)	15.2 (6.7)	12.9 (7.1)	15.9 (6.1)	16.2 (6.0)	14.0 (6.0)
RCFT recognition	19.6 (2.2)	19.7 (2.2)	19.2 (2.2)	20.1 (1.8)	20.2 (1.9)	19.6 (1.3)
Frontal/executive functions						
COWAT animal	15.9 (4.8)	16.1 (4.9)	14.7 (4.3)	16.8 (4.3)	16.7 (4.4)	17.3 (4.2)
COWAT phonemic total	27.2 (11.8)	27.6 (11.8)	25.4 (12.0)	29.2 (10.9)	29.0 (11.1)	30.0 (9.8)
K-CWST color reading	87.1 (21.2)	88.6 (21.2)	80.7 (20.3)	89.9 (19.8)	91.6 (19.1)	81.1 (21.7)
DSC	53.1 (19.5)	54.2 (19.8)	48.3 (17.1)	57.5 (19.7)	58.6 (19.6)	51.9 (19.6)
K-TMT-E-A time, s	24.6 (13.1)	24.1 (13.3)	26.9 (11.9)	22.0 (10.5)	21.7 (10.8)	23.0 (8.7)
K-TMT-E-B time, s	56.5 (54.1)	55.2 (54.3)	62.7 (53.1)	41.6 (30.7)	39.6 (30.9)	51.6 (27.7)
Others						
K-MMSE	28.1 (1.8)	28.2 (1.8)	27.7 (1.5)	28.4 (1.6)	28.4 (1.5)	28.5 (2.3)

N, number; *APOE* ε4, Apolipoprotein E; Aβ, amyloid-β; K-BNT, the Korean version of the Boston Naming Test; CDT, the Clock Drawing Test; RCFT, the Rey-Osterrieth Complex Figure Test; SVLT-E, the Seoul Verbal Learning Test-Elderly's version; COWAT, the Controlled Oral Word Association Test; K-CWST, the Korean Color Word Stroop Test; DSC, Digit Symbol Coding; K-TMT-E-A, the Korean Trail Making Test-Elderly’s version part A; K-TMT-E-B, the Korean Trail Making Test-Elderly’s version part B; K-MMSE, the Korean Mini-Mental State Examination.

**p < 0.05 between Aβ− and Aβ+ in both sets.

^a^Values are presented as mean (standard deviation) or number (%).

^b^The Independent sample t-test was used for continuous variables, and the chi-square test was used for categorical variables.

^c^Analysis of covariance was conducted as a statistical analysis to see the difference in test scores of each group. Age, education, and sex were adjusted as covariates in the analysis.

^d^*APOE* ε4 genotyping: development set N = 395; validation set N = 167.

### MIMIC model for latent mean analysis

The confirmatory factor analysis (CFA) model was successfully validated to control measurement errors. Accordingly, error covariance was added between the residual variances associated with the Seoul Verbal Learning Test-Elderly’s version (SVLT-E) immediate and delayed recalls and the Rey-Osterrieth Complex Figure Test (RCFT) immediate and delayed recalls. The CFA model with added error covariance fit the data well (χ^2^ = 212.181, *df* = 78, *p* < 0.001; RMSEA = 0.064; CFI = 0.957; TLI = 0.942; SRMR = 0.056). All factor loadings in the model were significant between 0.49 and 0.89. Next, a latent mean difference between Aβ+ and Aβ− for each cognitive domain was verified. The latent mean model fit the data well (χ^2^ = 359.481, *df* = 128, *p* < 0.001; RMSEA = 0.065; CFI = 0.944; TLI = 0.919; SRMR = 0.048). The result revealed that the differences between the Aβ+ and Aβ− groups in attention, visuospatial function, and language function were not significant, but the latent means in the Aβ+ group were significantly lower than the Aβ− group in the three domains of verbal memory, visual memory, and executive functions (Table 2).

**Table 2:** Latent mean difference between amyloid positive and negative groups for neuropsychological domains.

Neuropsychological domains	Estimate	SE	Bias-corrected bootstrap percentile (95% CI)
Amyloid positivity → Attention	0.040	0.114	(− 0.168, 0.282)
Amyloid positivity → Visuospatial function	0.159	0.375	(− 0.657, 0.814)
Amyloid positivity → K-BNT	− 0.017	0.782	(− 1.961, 1.122)
Amyloid positivity → Verbal memory	− 0.809	0.345	(− 1.662, − 0.213)
Amyloid positivity → Visual memory	− 1.449	0.724	(− 3.004, − 0.233)
Amyloid positivity → Frontal EF	− 0.440	0.213	(− 1.109, − 0.094)

Sex, education, and age were adjusted as covariates in the analyses.

K-BNT, the Korean Boston Naming Test; Frontal EF, frontal executive functions; SE, standard error; 95% CI, 95% Confidence Interval.

### MANCOVA

Based on the results above, further statistical analyses were conducted for each neuropsychological assessment within the episodic memory and executive functions. Multivariate analysis of covariance (MANCOVA) was used to see the score differences of the tests under episodic memory and executive functions between the Aβ+ and Aβ− groups when sex, education, and age were controlled. The result of the MANCOVA is shown in Table 3. Few neuropsychological subtests showed meaningful differences between the groups. Primarily, we set the level of significance at 0.1. Regarding episodic memory, the SVLT-E delayed recall showed a difference in score between the Aβ+ and Aβ− groups (*F*(1, 418) = 3.666, *p* = 0.056). For the RCFT, the Aβ+ group performed worse, not only on the delayed recall (*F*(1, 418) = 4.036, *p* = 0.045), but also on the immediate recall (*F*(1,418) = 2.898, *p* = 0.089). However, due to the extremely high correlation between the two subtests (*r* = 0.935), we considered that it would be reasonable to use only one subset in our composite model. Based on the clinical and statistical significance, the RCFT delayed recall was favored over the RCFT immediate recall for the PASC. In terms of executive functions, the Korean-Color Word Stroop test (K-CWST) color reading (*F*(1, 418) = 4.745, *p* = 0.030) and the Controlled Oral Word Association Test (COWAT) animal naming showed worse performance in the Aβ+ group compared to that in the Aβ− group (*F*(1, 418) = 3.152, *p* = 0.077).

**Table 3:** MANCOVA with neuropsychological tests in Memory and Executive Functions.

	Wilks' Lambda	Mean Square	F	*p* value
**Verbal memory**	0.991		1.274	0.283
SVLT-E immediate recall		31.149	2.007	0.157
SVLT-E delayed recall*		11.700	3.666	0.056
SVLT-E recognition		4.760	1.564	0.212
**Visual memory**	0.989		1.595	0.190
RCFT immediate recall*		110.640	2.898	0.089
RCFT delayed recall*		132.980	4.036	0.045
RCFT recognition		5.195	1.263	0.262
**Executive functions**	0.981		1.602	0.161
COWAT animal*		57.491	3.152	0.077
COWAT phonemic total		40.766	0.416	0.560
K-CWST color reading*		1,305.356	4.745	0.030
DSC		436.424	2.303	0.178
K-TMT-E-B time		100.874	0.052	0.843

Sex, education, and age were adjusted as covariates in the analyses.

RCFT, the Rey-Osterrieth Complex Figure Test; SVLT-E, the Seoul Verbal Learning Test-Elderly's version; COWAT, the Controlled Oral Word Association Test; K-CWST, the Korean Color Word Stroop Test; DSC, Digit Symbol Coding; K-TMT-E-B, the Korean Trail Making Test-Elderly’s version part B.

*p < 0.1

### Development of the preclinical amyloid sensitive composite (PASC) model

**Figure 1:**
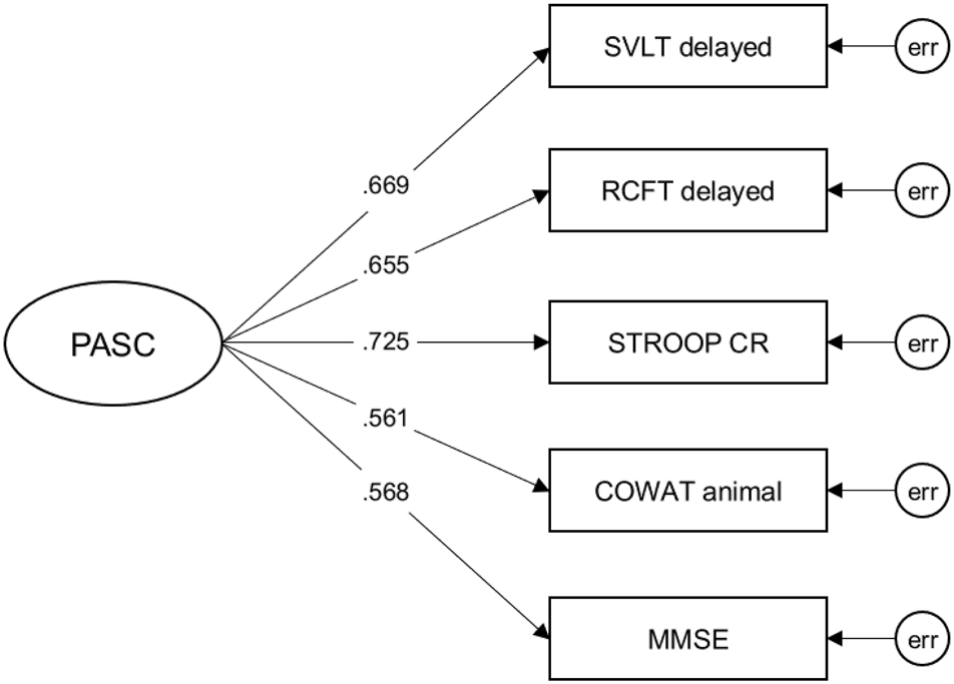
The CFA model of the PASC. Abbreviations: CFA, Confirmatory Factor Analysis; PASC, the Preclinical Amyloid Sensitive Composite; SVLT delayed, the Seoul Verbal Learning Test-Elderly's version delayed recall; RCFT delayed, the Rey-Osterrieth Complex Figure Test delayed recall; STROOP CR, the Stroop color reading test; COWAT animal, the Controlled Oral Word Association Test animal naming; MMSE, the Mini-Mental State Examination; err, error.

**Figure 2:**
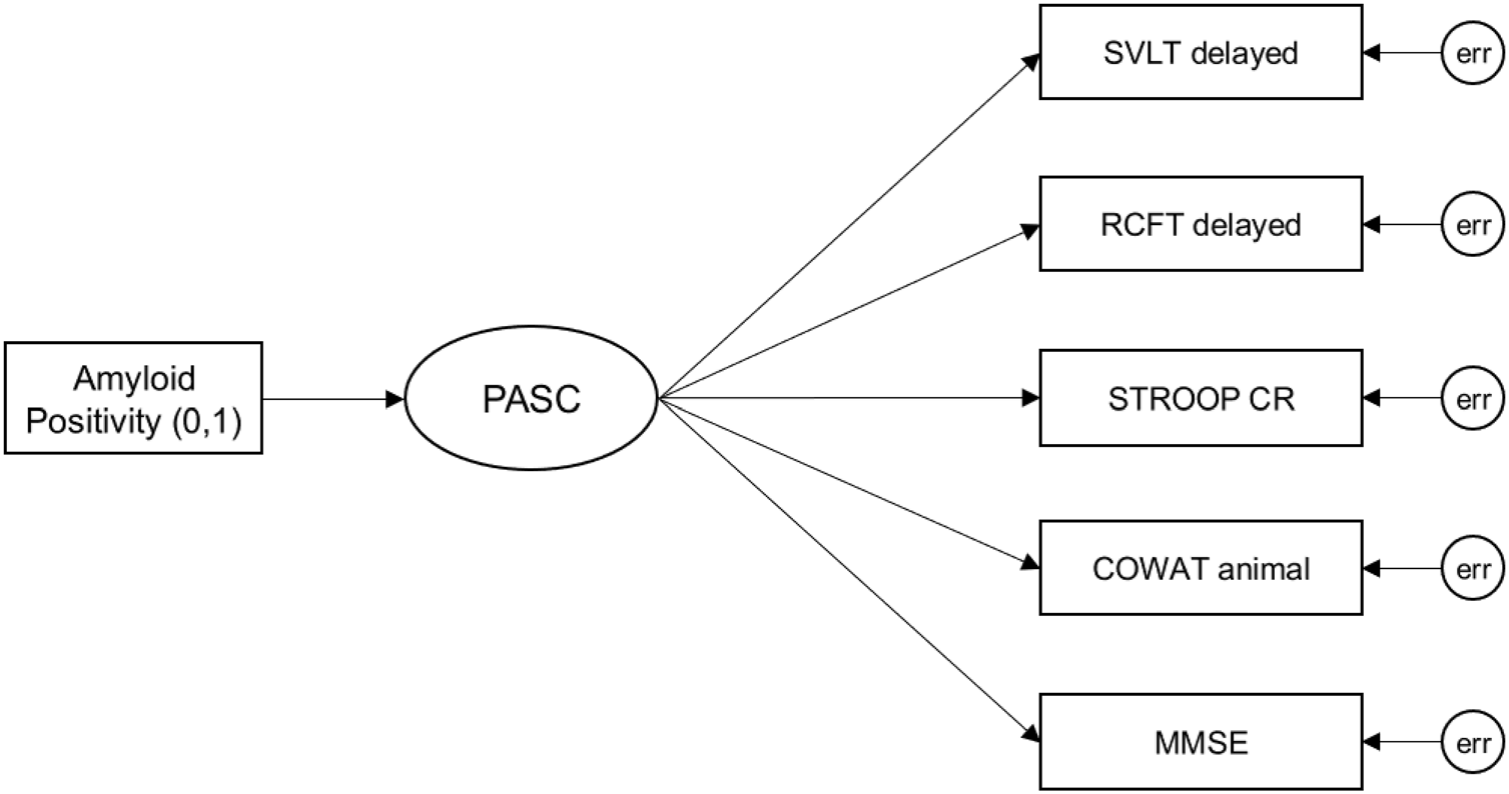
The MIMIC model of the PASC for latent mean comparison between Aβ+ and Aβ− cognitively normal participants. For amyloid positivity, 0 and 1 indicate Aβ− and Aβ+ respectively. Age, education, and *APOE* ε4 were adjusted as covariates. Abbreviations: MIMIC, Multiple-Indicators Multiple-Causes; PASC, the Preclinical Amyloid Sensitive Composite; Aβ, amyloid-β; *APOE* ε4, Apolipoprotein E; SVLT delayed, the Seoul Verbal Learning Test-Elderly's version delayed recall; RCFT delayed, the Rey-Osterrieth Complex Figure Test delayed recall; STROOP CR, the Stroop color reading test; COWAT animal, the Controlled Oral Word Association Test animal naming; MMSE, the Mini-Mental State Examination; err, error.

Based on the MANCOVA results and the literature, the following 5 tests were finally selected: the SVLT-E delayed recall; the RCFT delayed recall; the K-CWST color reading; the COWAT animal naming; and the Korean Mini-Mental State Examination (K-MMSE). The K-MMSE was added for examining global cognition. The PASC CFA model presented a good fit with the data (χ^2^ = 4.757, *df* = 5, *p* = 0.933; RMSEA < 0.001; CFI = 1.000; TLI = 1.001; SRMR = 0.014). All factor loadings in the model were significant between 0.56 and 0.73 (Fig. 1). The MIMIC model was used to ensure that the PASC distinguished between Aβ+ and Aβ− (Fig. 2). Our MIMIC model for the PASC fit the data well (χ^2^ = 56.526, *df* = 21, *p* < 0.001; RMSEA = 0.063; CFI = 0.955; TLI = 0.936; SRMR = 0.036). The result showed that the latent mean in the Aβ+ group was significantly lower than the Aβ− group (*t* = -2.340, *p* = 0.019) (Table 4).

**Table 4:** Latent mean difference between amyloid positive and negative groups for PASC.

Latent mean difference	Estimate	SE	Bias-corrected bootstrap percentile (95% CI)
Amyloid positivity → PASC	− 0.345*	0.147	(− 0.641, − 0.049)

Education, age, and *APOE* ε4 were adjusted as covariates in the analyses.

PASC, the Preclinical Amyloid Sensitive Composite; SE, Standard Error; 95% CI, 95% Confidence Interval.

*p < 0.05.

### Calculation of the PASC score

In order to create the composite score, we implemented the principal component analysis (PCA) with the z-scores of the 5 tests. As a result, the following composite equation was generated:

$${\text{PASC}} = .70\left( {SVLT delayed z} \right) + .61\left( {RCFT delayed z} \right) + .67\left( {Stroop CR z} \right) + .55\left( {COWAT animal z} \right) + .58\left( {MMSE z} \right)$$

**Figure 3:**
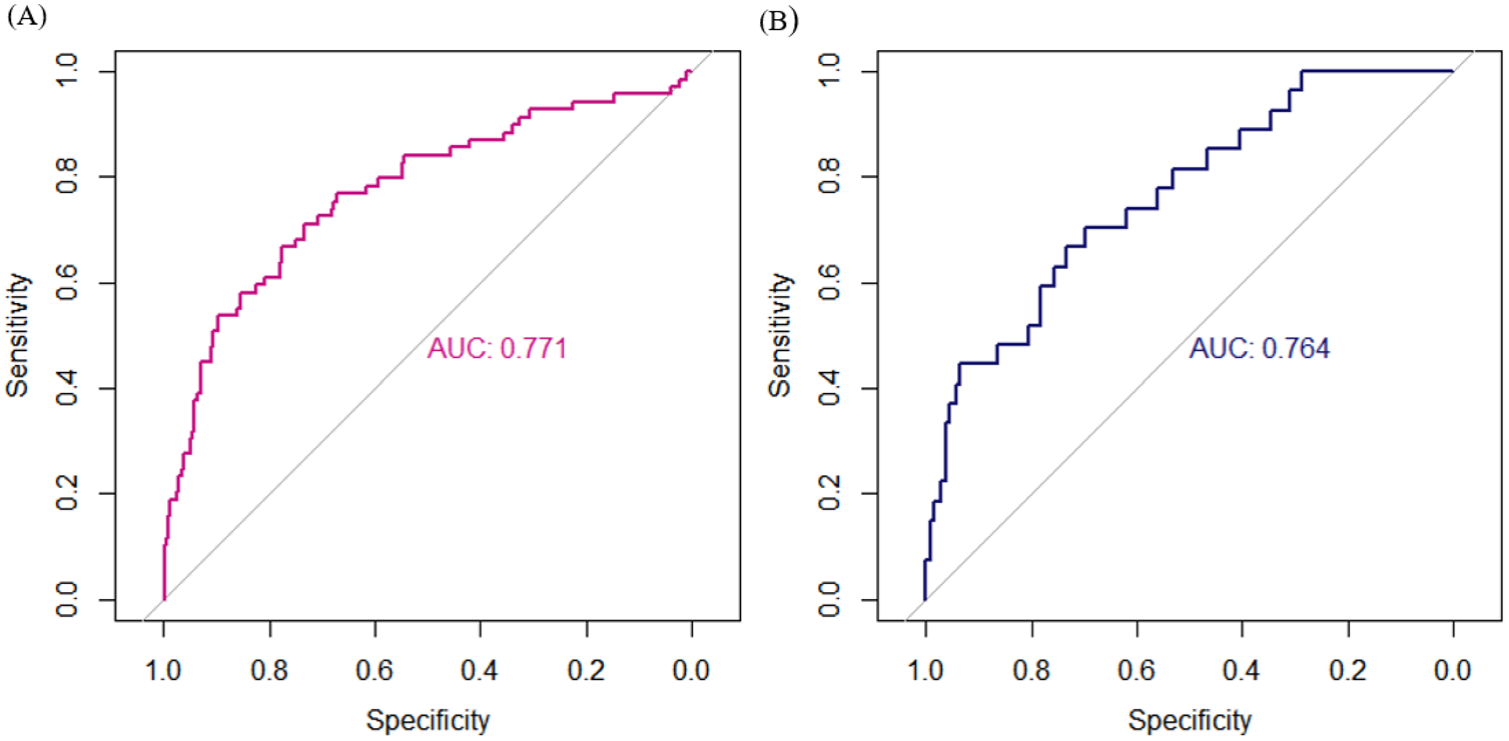
The ROC curve analysis of the PASC score, age, sex, education, and *APOE* ε4 in the development (**A**) and validation (**B**) sets. Abbreviations: ROC, Receiver Operating Characteristic; PASC, the Preclinical Amyloid Sensitive Composite; *APOE* ε4, Apolipoprotein E; AUC, Area Under the Curve.

The receiver operating characteristic (ROC) curve analysis presented a decent accuracy for the PASC score when applied with age, sex, education, and *APOE ε*4 to distinguish between Aβ+ and Aβ− (AUC = 0.771; 95% CI = 0.704–0.837) (Fig. 3a, Table 5). The sensitivity (71%) and specificity (73.5%) were optimal for distinguishing between Aβ+ and Aβ−, and the Youden index was 0.445.

**Table 5:** Accuracy of the ROC curve analysis of the PASC score, age, sex, education, and *APOE* ε4 in the development and validation sets.

	AUC	95% CI	Sensitivity (%)	Specificity (%)
Development set	0.771	0.704–0.837	71.0	73.5
Validation set	0.764	0.667–0.860	70.4	69.8

ROC, Receiver Operating Characteristic; PASC, the Preclinical Amyloid Sensitive Composite; *APOE* ε4, Apolipoprotein E; AUC, Area Under the Curve; 95% CI, 95% Confidence Interval.

### External validation

The demographic and neuropsychological features of the validation sample are described in Table 1. The overall mean age of the validation set was comparable with that of the development set (69.4 ± 8.2 years vs. 69.9 ± 8.1, *p* = 0.494). Similarly, the years of education in the validation set were not different from those in the development set (12.0 ± 4.4 vs. 11.8 ± 4.8, *p* = 0.527). The proportion of female in the validation sample also did not differ from that in the development sample (60.3% vs. 63.1%, *p* = 0.519). Moreover, the rate of Aβ+ of the validation sample was similar to that of the development sample (16.2% vs. 17.7%, *p* = 0.650). The proportion of *APOE ε*4 carriers in the validation set was also at comparable levels to that in the development set (22.8% vs. 24.6%, *p* = 0.648). Within the validation sample, the Aβ+ group was significantly older than the Aβ− group (73.7 years vs. 68.6 years, *p* < 0.05). The Aβ+ group also displayed a higher percentage of *APOE ε*4 carriers compared with the Aβ− group (51.9% vs. 17.1%, *p* < 0.001). However, the two groups did not significantly differ in education level and proportion of female participants. The PASC CFA model exhibited a good fit with the validation sample (χ^2^ = 2.338, *df* = 5, *p* = 0.801; RMSEA < 0.001; CFI = 1.000; TLI = 1.028; SRMR = 0.016). The MIMIC model for the PASC showed a fair fit to the validation sample (χ^2^ = 49.870, *df* = 21, *p* < 0.001; RMSEA = 0.088; CFI = 0.917; TLI = 0.882; SRMR = 0.051). The accuracy of the PASC score when applied with age, sex, education, and *APOE ε*4 for distinguishing between Aβ+ and Aβ− was adequate (AUC = 0.764; 95% CI = 0.667–0.860). The sensitivity and specificity were also optimal when the Youden index was 0.402 (sensitivity = 70.4%; specificity = 69.8%) (Fig. 3b, Table 5). The results of the ROC curve analysis in the external validation set were comparable to those in the development set (Table 5).

## Discussion

We investigated the distinct neuropsychological features of Aβ+ CN elderlies in a carefully phenotyped, CN cohort that underwent detailed neuropsychological tests, MRI, and amyloid PET scans with the standardized protocols. Accordingly, there were several significant neuropsychological findings in this study. First, the MIMIC model found the latent mean difference between the Aβ+ and Aβ− groups in the domains of verbal memory, visual memory, and executive functions. Furthermore, MANCOVA showed that the Aβ+ group performed worse in the SVLT-E delayed recall, the RCFT delayed recall, the K-CWST color reading, and the COWAT animal naming within the three cognitive domains. The PASC model that we developed using the result of MANCOVA and the MMSE presented a good fit with the data. Finally, the accuracy of the PASC score when applied with age, sex, education, and *APOE ε*4 for distinguishing between Aβ+ and Aβ− was adequate (AUC = 0.764; 95% CI = 0.667–0.860) in the external validation set (N = 179). Our results, therefore, suggested that the PASC might contribute to decreasing financial loss due to screen failures in preclinical AD clinical trials and facilitating more prevention trials subsequently.

The demographic profile of our participants was extremely close to that of the previously reported Asian society profile. The CN Aβ+ percentage in Asian countries is known to be lower than that in western countries. The percentage of CN amyloid positivity in the Asian population ranged between 18 and 25% according to the Korean Brain Aging Study for the Elderly Diagnosis and Prediction of Alzheimer’s disease (KBASE) and Japanese ADNI (J-ADNI)^13,14^. On the other hand, the western population, represented by ADNI, was reported to range approximately from 25 to 45% Aβ positivity rate^15,16^. Our study exhibited approximately 18% Aβ positivity in the 423 CN individuals, which was in line with that in the Asian population. The discrepancy between our results and that of the western society may be explained by the differences in the frequency of *APOE ε*4 and the age of the study participants. Our cohort seemed to have a lower percentage of *APOE ε*4 (23%) than that reported by ADNI (27%)^17^. Moreover, the younger age of our cohort (mean, 69.9 years) compared to that of the ADNI CN individuals (mean, 75.8 years) may have affected the lower rate of amyloid positivity^17^. Regardless of these disparities, the *APOE ε*4 rate and the age of our cohort were still at comparable levels to J-ADNI’s *APOE ε*4 rate (24%) and CN individuals’ ages (mean, 67.9)^13^.

Our major finding was that the Aβ+ CN individuals presented a lower performance in verbal memory, visual memory, and executive functions compared to Aβ− CN, which was generally consistent with the findings of previous meta-analyses. In terms of memory, there has been a consensus that episodic memory has a strong association with Aβ burden^18–20^. In our study, delayed recall task of both verbal and visual memory tests especially stood out as the performance difference between Aβ+ and Aβ− seem to be more prominent than immediate or recognition tasks. This result is not surprising because previous studies with CN or MCI also suggested that using delayed recall may be good predictor of Aβ positivity^21,22^. Unlike episodic memory, the results regarding executive functions in the previous studies are not entirely consistent. A recent meta-analysis suggested a significant difference in executive function^19^, while two others showed either a small effect size or a weak association with Aβ burden^18,20^. This may be because the previous studies did not consider the effects of measurement errors that could impact the individual test scores. However, applying a factor analysis with the latent variables, we controlled for the measurement errors from each test score for more precise measurement of the corresponding cognitive function.

In the present study, we also developed the PASC that is sensitive to the subtle cognitive differences in CN based on amyloid positivity. The PASC comprises the SVLT-E delayed recall, the RCFT delayed recall, the COWAT animal naming, and the K-CWST color reading, which were found to be significantly different between the two CN groups from the three cognitive domains, and the K-MMSE. We included the K-MMSE in the PASC because the Mini-Mental State Exam (MMSE) is a practical neuropsychological test to examine individual cognitive function holistically^23^. Global cognition was previously reported to be associated with amyloidosis^7,24–26^, and has been considered to help with early identification of dementia risk and further cognitive decline^27^. Furthermore, other composite scores such as the Preclinical Alzheimer Cognitive Composite (PACC) and the Alzheimer’s Prevention Initiative Composite Cognitive Test Score (APCC) include the MMSE for global functioning and orientation status^28–30^. In this regard, we included the MMSE for the convenience of harmonization in the future international collaboration. In fact, the PASC may seem similar to the PACC^29^. However, the PACC has been mainly applied to track cognitive changes in preclinical AD over time^25,31^, whereas the PASC investigated the cognitive difference between Aβ+ CN and Aβ− CN cross-sectionally.

Although AD pathology progression involves the deterioration of multiple cognitive domains, there are a few benefits to observe cognitive differences in CN individuals with a single composite that is a unidimensional outcome. First, it allows comprehensive yet precise cognitive assessment particularly in preclinical AD. Currently, the MMSE^23^ and the Clinical Dementia Rating (CDR)^32^ are commonly used to assess individual cognitive function holistically. However, they often display ceiling effects in CN individuals^33,34^. Therefore, they are not quite sensitive measures for CN individuals. Furthermore, the ratings of the CDR primarily rely on clinicians’ judgments following patient and caregiver interviews. In other words, bias is rarely avoidable in the CDR. As a result, there is a need for a novel and reliable measure to holistically assess cognitive function specific to preclinical AD, and we expect that the PASC can meet the need. Another advantage of obtaining a unidimensional composite is that it induces a more precise result in the outcome measurement. Compared to using multi-outcomes, using a single outcome usually yields lower background noise in the measurement, which derives a lower risk of Type-I error^11,28^. Therefore, applying a single primary outcome has better reliability and sensitivity especially in terms of detecting subtle cognitive differences in preclinical AD.

The major strength of our study is that we considered measurement errors in the test scores when we implemented the analyses. Another strength is the large sample size of the CN cohort who underwent amyloid PET. In spite of these strengths, there are a few limitations to our study. First, the participants went through different types of PET ligands. The variety of the tracers may have affected the visual reads of amyloid deposition. However, this limitation can be somewhat alleviated by the high correlations among the different ligands^35,36^. Second, we did not explore the clinical effects of the PASC. Future studies with clinical impacts of the PASC on other biomarkers like tau or cortical atrophy may be recommended. Another limitation is that our study used dichotomized variable of amyloid burden. The issue about dichotomization of amyloid deposition has been constantly questioned as there were several studies showing longitudinal cognitive decline related to subthreshold amyloid in Aβ− CN individuals^37–39^. In future studies, continuous measure of amyloid burden may be used to embrace the issue of subthreshold amyloid. Also, longitudinal studies using the PASC may be needed to examine the clinical applicability related to the issue.

Our study created the PASC which is a sensitive cognitive composite score for Aβ+ in CN elderly individuals, subsequent to investigating some distinct cognitive features of Aβ+ in CN elderly individuals. The PASC, which employed significant tests in episodic memory and executive functions, along with the global cognitive measure of the K-MMSE, showed adequate accuracy when it was applied with age, sex, education, and *APOE ε*4. Therefore, we expect the PASC to be applied potentially into diverse forms of studies such as trial ready registries^40,41^ and to contribute to decreasing financial loss due to screen failures and facilitating more prevention trials subsequently. Moreover, given that the cognitive tests that reflect the characteristics of early preclinical AD are anticipated to reflect the later cognitive change, we expect the PASC to be used for monitoring of disease progression or therapeutic efficacy.

## Methods

### Study participants

A total of 423 CN participants were recruited from September 2015 to December 2018 at the Samsung Medical Center in Seoul, South Korea. All the participants met the following criteria to be qualified as CN: (a) the K-MMSE 24 or above -1.5 standard deviation (SD) from the age-, sex-, and education-adjusted norms if the education period was less than 9 years; (b) above -1 SD from the age-, sex-, and education-adjusted norms on the delayed recall of the SVLT-E; (c) above -2 SD from the age-, sex-, and education-adjusted norms on the Korean version of the Boston Naming Test (K-BNT), the RCFT copy, and the K-CWST color reading; and (d) an absence of other neurological disorders. The screenings were conducted by trained clinicians and neuropsychologists. Brain MRI confirmed the absence of structural lesions, including territorial cerebral infarction, brain tumors, hippocampal sclerosis, vascular malformation, and cerebral amyloid angiopathy (CAA).

The external validation sample involved 91 CN participants who were recruited from December 2018 to April 2020 at the Samsung Medical Center and 88 CN participants who were recruited from May 2017 to April 2020 at Gangnam Severance Hospital. None of the participants in the external validation sample was included in the original study sample.

Written informed consents were obtained from each participant. This study was approved by the Institutional Review Board at the Samsung Medical Center. All methods were implemented in accordance with the approved guidelines.

### ^18^F-labeled amyloid PET acquisition and analysis

A total of 423 CN participants underwent ^18^F-labelled amyloid PET; 219 underwent ^18^F-florbetaben PET, 203 underwent ^18^F-flutemetamol PET, and 1 underwent ^18^F- florbetapir PET scanning at the Samsung Medical Center. The scanning was performed using a Discovery Ste PET/CT scanner (GE Medical Systems, Milwaukee, WI, USA) with a 3D scanning mode that examined 47 slices of 3.3 mm thickness spanning the entire brain. Prior to a 20-min emission PET scan with dynamic mode consisting of 4 × 5 min frames, 311.5 MBq ^18^F-florbetaben, 197.7 MBq ^18^F-flutemetamol, and 370 MBq ^18^F-florbetapir were injected. The scan was performed 90 min after the injection. 3D PET images were reconstructed in a 128 × 128 × 48 matrix with a 2 × 2 × 3.27 mm voxel size using the ordered-subsets expectation–maximization algorithm (^18^F-florbetaben, iteration = 4 and subset = 20; ^18^F-flutemetamol, iteration = 4 and subset = 20; ^18^F-florbetapir, iteration = 4 and subset = 16).

Visual assessment was done by three experienced raters (two nuclear medicine doctors and one neurologist) who were blinded to patient information, and the assessment was dichotomized as Aβ+ or Aβ− using visual reads. The visual assessments for ^18^F-florbetaben PET, ^18^F-flutemetamol PET, and ^18^F-florbetapir PET were performed with the scoring system that was used in the previous studies^42–46^. Inter-rater agreement was excellent for both FBB (Fleiss k = 0.86) and for FMM (Fleiss k = 0.78). After the raters individually rated, we determined the final visual positivity based on the majority visual reading result. Also, both FBB and FMM showed the high concordance rates between visual assessment and SUVR cutoff categorization for Aβ deposit (93.5% in FBB and 91.6% in FMM). The raters had successfully completed the electronic training program provided by the manufacturer to be qualified for the visual assessment.

### Neuropsychological assessments

The second edition of the Seoul Neuropsychological Screening Battery (SNSB-II) was administered to all the participants to assess their cognitive functions^47,48^. The SNSB-II was standardized on 1,067 CN elderly individuals in South Korea^48^. The normative data for the individual neuropsychological test was established based on a representative of South Korean population with age between 45 and 90 and the education level over 18 years. In our study, we used the following tests that are included in the SNSB-II: Digit Span Test (DST) forward and backward for attention; the K-BNT for language; the Clock Drawing Test (CDT) and the RCFT for visuospatial function and visual memory; the SVLT-E for verbal memory; and phonemic and semantic COWAT, K-CWST, Digit Symbol Coding (DSC), and the Korean Trail Making Test-Elderly’s version (K-TMT-E) for executive functions. The RCFT involved copying, immediate recall, 20-min delayed recall, and recognition tests. Similarly, the SVLT-E was composed of immediate recall trials, delayed recall, and recognition tests. In addition to the tests mentioned above, the K-MMSE was also used for the global mental state assessments of the participants^49^.

### Statistical analyses

Demographic characteristics were compared between the Aβ+ and Aβ− groups using the independent sample t-test if the variables were continuous and the chi-square test if the variables were categorical.

**Figure 4:**
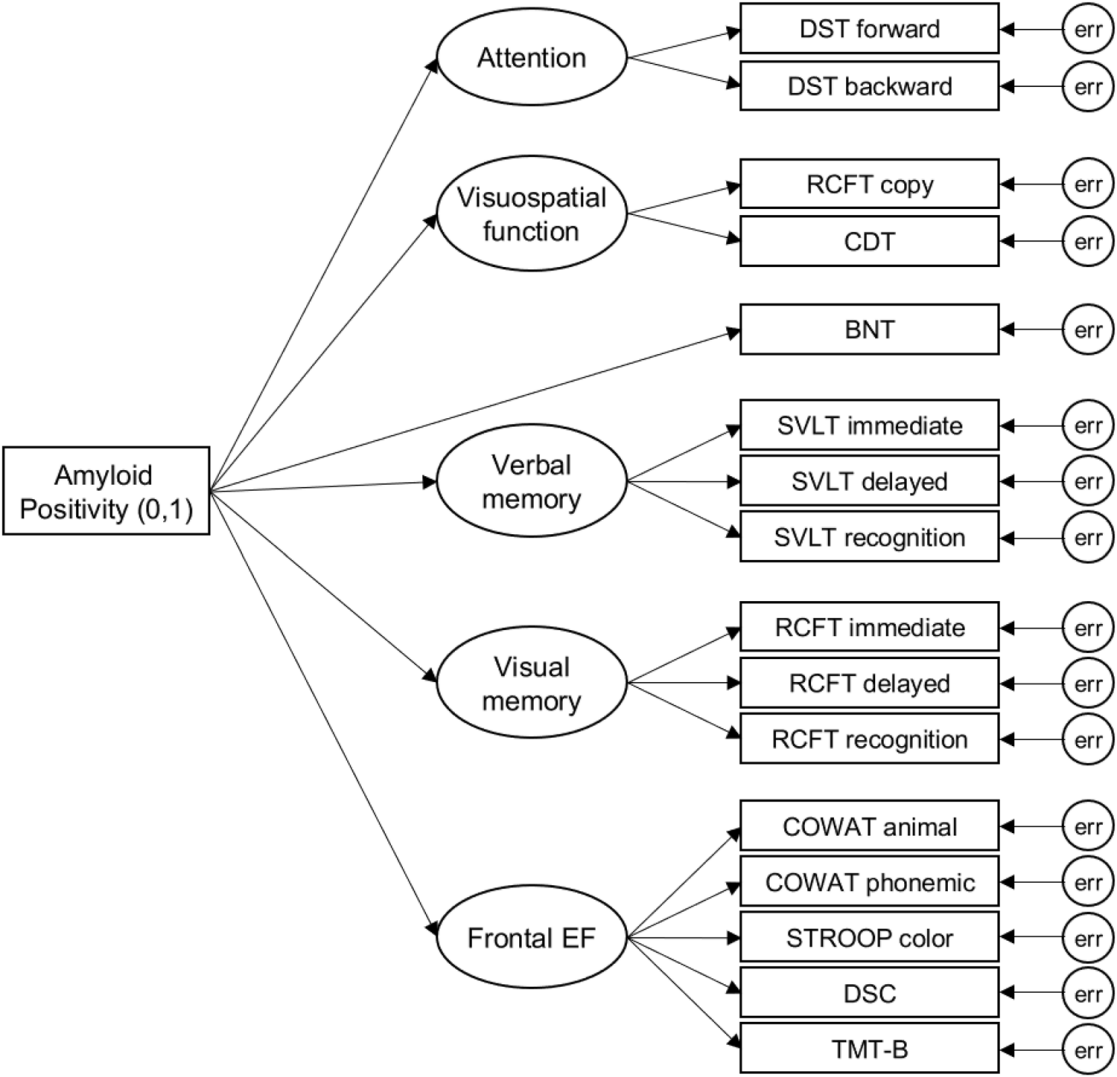
The MIMIC model of the SNSB-II. The model was created for latent mean comparisons in the cognitive domains between Aβ+ and Aβ−. For amyloid positivity, 0 and 1 indicate Aβ− and Aβ+ respectively. Age, sex, and education were adjusted as covariates. Abbreviations: MIMIC, Multiple-Indicators Multiple-Causes; SNSB-II, the second edition of the Seoul Neuropsychological Screening Battery; Frontal EF, Frontal Executive Functions; DST, Digit Span Test; RCFT copy, the Rey-Osterrieth Complex Figure Test copy figure; CDT, the Clock Drawing Test; BNT, the Boston Naming Test; SVLT immediate, the Seoul Verbal Learning Test-Elderly's version immediate recall; SVLT delayed, the Seoul Verbal Learning Test-Elderly's version delayed recall; SVLT recognition, the Seoul Verbal Learning Test-Elderly's version recognition; RCFT immediate, the Rey-Osterrieth Complex Figure Test immediate recall; RCFT delayed, the Rey-Osterrieth Complex Figure Test delayed recall; RCFT recognition, the Rey-Osterrieth Complex Figure Test recognition; COWAT, the Controlled Oral Word Association Test; STROOP color, the Stroop color reading test; DSC, Digit Symbol Coding; TMT-B, the Trail Making Test-Elderly’s version part B; err, error.

CFA was yielded to validate the structure of the five cognitive domains. CFA is one of the multiple forms of SEM, which confirms whether a pre-specified factor structure fits the data well^11^. We validated the CFA model for the neuropsychological test battery to control for measurement errors. The tests included in each cognitive domain were the same as those described earlier, and the language domain consisted of a single test score. The subtests of the SVLT-E and the RCFT in the memory domain were measured respectively using the same method. Therefore, it was considered acceptable to add an error covariance between the residual variances associated with the SVLT-E immediate and delayed recalls and the RCFT immediate and delayed recalls. Since our factor structure included both reflective and causal indicators, we used the MIMIC model to compare the latent means in the cognitive domains between the Aβ+ and Aβ− groups (Fig. 4).

MANCOVA was performed to see if any neuropsychological tests showed a significant difference between the two groups. Since the measurement errors were not treated in the MANCOVA, we deliberately set the cutoff for significance to be less conservative in order to increase the power and reduce the risk of type II errors. Thus, the tests with *p*-value < 0.1 were selected to be included in the composite model. The MIMIC model was repeated to identify whether these tests were sensitive to differences between Aβ+ and Aβ− in CN elderlies as a composite. For the PASC score equation, the PCA was used to obtain the weight for each test score. Accuracy, sensitivity, and specificity of the PASC score combined with age, sex, education, and *APOE ε*4 for distinguishing between Aβ+ and Aβ− were tested by ROC analysis.

The CFA and the MIMIC models of the PASC were validated in the cross-validation sample. The accuracy for distinguishing between Aβ+ and Aβ− of the PASC score combined with age, sex, education, and *APOE ε*4 was investigated by the ROC curve analysis in the external validation sample.

Raw score of each test was used in the statistical analyses for development of the PASC model. Z-scores were used to compute the PASC score. The K-TMT-E part B was log-transformed for accuracy of the estimate due to its large range (0–300) and non-normality. Multiple imputation and full information maximum likelihood estimations were used to treat missing values.

IBM SPSS (version 25.0, SPSS Statistics/IBM Corp, Armonk NY, USA) was used for the statistical analyses. For comparisons of latent means between the groups, maximum likelihood estimation was analyzed by Mplus (version 8.0)^50^. However, due to the violation of normality, bias-corrected bootstraps were performed together.
